# Comparative Analysis of Mathematical Models and App-Based Measurement for Estimating the Cutaneous Wound Areas of Captive Asian Elephants

**DOI:** 10.7759/cureus.65533

**Published:** 2024-07-27

**Authors:** Thyagaraj Giggin, Kurisinkal D Martin, Syam K Vebugopal, Kundukulam S Anil, Arayinkaravattu R Sreeranjini, Mulluparambil K Narayanan

**Affiliations:** 1 Department of Veterinary Surgery and Radiology, Kerala Veterinary and Animal Sciences University, Kerala, IND; 2 Department of Veterinary and Animal Sciences, Kerala Veterinary and Animal Sciences University, Kerala, IND; 3 Department of Livestock Production and Management, College of Veterinary and Animal Sciences, Kerala Veterinary and Animal Sciences University, Kerala, IND; 4 Department of Veterinary Anatomy and Histology, Kerala Veterinary and Animal Sciences University, Kerala, IND

**Keywords:** non-contact measurements, animal welfare, captive asian elephants, mobile applications, smartphones, digital planimetry, wound measurements, skin injuries

## Abstract

Objective

To evaluate the variation in the area estimation under different mathematical calculations against measurement by a smartphone application in estimating the cutaneous wound areas in captive Asian elephants.

Methods

The study was conducted on captive Asian elephants (Elephas maximus) with cutaneous wounds reported to Veterinary Hospitals of Kerala Veterinary and Animal Sciences University and elephant camps within and outside Kerala state (mostly southern states of India, namely, Kerala and Tamil Nadu) over the period September 2019 to October 2022. Thirty-five clinical cases diagnosed with skin wounds of different aetiologies at various parts of the body were subjected to measurement, and 111 measurements were taken using a smartphone application, Imito Measure (Imito AG, Zurich, Switzerland). Based on the outer wound perimeters hand-marked on the mobile screen over the image taken, Imito Measure calculated the length, width, perimeter, and area. The length and width measurements from this were applied to four mathematical models of wound measurements. Wound surface area calculations were further done by these models and were compared.

Results

The observed results indicated no significant difference between the five methods of area measurement in all the studied cases since the P > 0.05.

Conclusion

The findings revealed no significant difference between the five techniques of wound area measurement. From the practical clinical utility point, the smartphone application has an edge over the mathematical methods in animals, especially captive Asian elephants, as it has the major advantage of being non-contact and thus addresses some major welfare concerns.

## Introduction

As the skin provides primary protection, the cutaneous wounds resulting from external violence are a common problem in elephants. The occurrence of cutaneous wounds in captive Asian elephants was previously recorded at 44% in Sri Lanka [1], 19.1% and 21.2% in two centers in Thailand [2], and 6.9% in North America [3]. In India, studies reported 16% [4], 39.72%, and 38.64% in two places [5]. In the past few years, numerous methods for assessing the area and volume of wounds have been published in the literature. Assessment methods must be accurate, reliable, and practical when analyzing treatment regimens in clinical work and research investigations. The delineation of the wound border, which is sometimes challenging to figure out, is one of the elements influencing wound measuring accuracy. Small motions can alter the look of a wound, and location on a curved area of the body can make estimating wound size difficult. Some wounds are also severely damaged, making it impossible to determine the volume. Lastly, wounds in places with thick soft tissue covering might produce complications due to physiological contraction [6].

Traditionally, wound measuring methodologies have relied on two-dimensional approaches. The ruler approach sometimes overestimates the wound size and could be more effective in big wounds with uneven borders, yet it is quick, simple, and affordable. The elliptical approach is simple and economical and may be used to measure ellipse-shaped wounds [7,8].

The planimetric approach is simple to master and an accurate and dependable method that considers body curvature [9]. Although digital planimetry is marginally more precise and reliable than manual planimetry, both approaches entail contact with the wound and thus pose the danger of wound contamination [10]. Manual planimetry is time-consuming because it involves the precise marking of wound edges onto the tracing sheet and determining the traced area by manually counting 1 mm^2^ in that area. Compared to the planimetric technique, the digital imaging method is similarly precise and trustworthy, and it is a non-contact approach, eliminating the possibility of wound contamination and pain during handling, a significant welfare concern in the case of animals [11].

The present study aims to evaluate the variation in the area estimation by different mathematical calculations against measurement by the smartphone application.

## Materials and methods

The study was conducted on captive Asian elephants (Elephas maximus) with cutaneous wounds reported to Veterinary Hospitals of Kerala Veterinary and Animal Sciences University and elephant camps within and outside Kerala state (mostly southern states of India, namely, Kerala and Tamil Nadu) over the period September 2019 to October 2022 after obtaining the necessary permissions from the concerned owner/authority before the commencement of the study.

All animals, irrespective of age and sex, that presented to the surgery outpatient unit were screened for the evaluation of wounds. Clinical cases belonging to private owners were attended to, followed by a request from the owner/custodian or after obtaining informed consent from the concerned person/authority.

Thirty-five clinical cases diagnosed with skin wounds of various etiologies were subjected to measurement, and 111 measurements were taken. The measurements such as length, width, area, and perimeter were determined using the mobile application, and wound images were taken.

Wound measurement by a smartphone application

The smartphone application ImitoMeasure (Imito AG, Zurich, Switzerland) was downloaded for free from the Google Play store to an Android smartphone, Samsung S-20 FE 5G (model: SM-G781B/DS). The calibrated marker for wound assessment comparison was received as a PDF file from the application's developers. The markers were printed onto 220 GSM paper, which was cut individually for use and disposal. To avoid inter-operator variations (variations between operators who may have different perceptions regarding the wound margin and variation in the level of expertise in using the mobile application), only one person measured the wound using this mobile application. The same phone was used throughout the study period to avoid device errors. The routine smartphone application and system updates were done during the entire study period.

Wound length, width, surface area, and perimeter measurement

After the debridement, wound lavage, and cleaning of the area, the calibrated marker was placed near the wound. The smartphone application, ImitoMeausre, was opened, and the smartphone's camera was aimed at the wound. The camera was aligned with a calibrated marker so that the camera detected the marker and allowed capture. The aligning was noted by the appearance of a green square around the calibrated marker and a message saying “marker detected” on the screen. The image of the wound was captured by clicking the " capture " button on the screen in the top left corner. Once the image was obtained, the wound perimeter was marked with a finger on the smartphone's screen. The markings appeared as green lines with dots on them. The drawings are completed by joining the end to its origin-the measurements are obtained by clicking the “measure” button on the screen. The result screen was with an image of the wound and its measurements. The wound length and width were green dotted lines with the lengthiest and widest widths. The application automatically calculates the wound surface area and perimeter when clicking the “measure” button.

Wound surface area calculation by other methods

Method (1): The exact length and width measurements of wounds calculated by the application were used for wound surface area calculations by mathematical methods (n=111) to avoid direct contact with a wound surface.

Method (2): A standard simple ruler method of wound surface area estimation for rectangular was done by multiplying the longest length by the widest width. In this method, the wound shape is considered as rectangular [12].

Method (3): A mathematical method of wound surface area estimation for ellipse was done by multiplying the longest length by the widest width with a factor of 0.785 or π/4. In this method, the wound shape is considered an ellipse [2].

Method (4): A mathematical method of wound surface area estimation optimized for human plantar ulcers was done by multiplying the longest length by the widest width with a factor of 0.73 [13].

Method (5): A mathematical method of wound surface area estimation optimized for venous ulcers in humans was done by multiplying the longest length by the widest width with a factor of 0.67 [13]. After this, the values are sorted as values with orthogonal length and width measurements (n=66) and values without orthogonal length and width measurements (non-orthogonal) (n=45).

Method (6): The calculations were done for orthogonal (n=66) as per the following formula [13]:

 (1) standard ruler method = longest length X orthogonal widest width

 (2) ellipse = longest length X orthogonal widest width X 0.785

 (3) optimized (p) = longest length X orthogonal widest width X 0.73

 (4) optimized (v) = longest length X orthogonal widest width X 0.67

The calculations are done for non-orthogonal (n=45) as per the following formula:

 (1) standard ruler method = longest length X widest width

 (2) ellipse = longest length X widest width X 0.785

 (3) optimized (p) = longest length X widest width X 0.73

 (4) optimized (v) = longest length X widest width X 0.67

Visual representation of over-estimation and underestimation

The over- and underestimation of all the measurements were graphically depicted for the standard ruler, elliptical, optimized (P), and optimized (V) measurement methods. A helpful visual representation of the relationship between two paired variables on the same scale is represented on the the Bland-Altman plot, which was used to compare the following variables: smartphone application measurement vs. standard ruler, smartphone application measurement vs elliptical, smartphone application measurement vs. optimized (P) and smartphone application measurement vs optimized (V).

## Results

Cutaneous wounds of elephants (n=111)

One hundred eleven observations from 35 wounds of captive Asian elephants at different stages were collected for study. The mean length was 8.45 ± 0.54 cm, the smallest was 0.64 cm, and the lengthiest was 33.94 cm (Table 1). The mean width was 4.23 ± 0.34 cm, the smallest was 0.23 cm, and the widest was 19.76 cm. The mean area was 34.61 ± 5.31 cm^2^, the smallest was 0.09 cm^2^, and the largest was 352.14 cm2. The mean perimeter was 21.83 ± 1.51 cm, where the smallest was 1.39 cm and the largest was 103.14 cm (Table 1).

**Table 1:** Mean, median, SD, SE, and mean ± SE.

Whole data (n=111)					
	Mean	Median	SD	SE	Mean ± SE
Length	8.454864865	7.36	5.681298486	0.53924499	8.45 ± 0.54 cm
Width	4.230900901	2.95	3.632824483	0.34481244	4.23 ± 0.34 cm
Area	34.60873874	15.64	55.69406136	5.3102204	34.61 ± 5.31 cm^2^
Perimeter	21.83396396	18.35	15.89293206	1.50849035	21.83 ± 1.51 cm

Cutaneous wounds with orthogonal measurements (n=66)

Sixty-six observations (59.46 % of total observations) from different wounds of captive Asian elephants at different stages were collected for this study. The mean length, width, area, and perimeter are presented in Table 2.

**Table 2:** Mean, median, SD, SE, and mean ± SE.

Orthogonal measurements (n=66)					
	Mean	Median	SD	SE	Mean ± SE
Length	8.501060606	8.365	4.675261944	0.575484963	8.50 ± 0.58 cm
Width	4.731363636	3.77	3.262563356	0.401593788	4.73 ± 0.40 cm
Area	35.66939394	22.51	48.75259715	6.001029872	35.67 ± 6.00 cm^2^
Perimeter	22.79015152	23.33	12.99571296	1.599661685	22.79 ± 1.59 cm
Standard	52.28987576	38.37035	69.19285012	8.517051087	52.29 ± 8.51 cm^2^
RMS error (Std)	51.37361897	46.25631122	20.58123252	2.533374598	51.37 ± 2.53 %
Elliptical	41.04755247	30.12072475	54.31638735	6.685885103	41.05 ± 6.69 cm^2^
RMS error (Eli)	18.88623725	14.8112043	16.08744663	1.980227792	18.88 ± 1.98 %
Optimized (P)	38.1716093	28.0103555	50.51078059	6.217447293	38.17 ± 6.22 cm^2^
RMS error (Opt-p)	12.18219026	6.885205916	13.67644997	1.683454618	12.18 ± 1.68 %
Optimized (V)	35.03421676	25.7081345	46.35920958	5.706424228	35.03 ± 5.71 cm^2^
RMS error (Opt-v)	9.816076114	7.632216017	9.713849977	1.195692277	9.82 ± 1.20 %

The Ac (elliptical) calculation in this dataset showed an overestimation of the area in 65 calculations (98.48%), and the average overestimation was 19.15 ± 1.99%. It is shown that underestimation in one analysis (1.52%) and average underestimation was 1.91% (Tables 3-4). However, the Ac optimized (P) calculation in this dataset shows an underestimation of the area in 16 out of 66 measures (24.24%). The average underestimation was 3.46 ± 0.64%. Fifty out of 66 calculations (75.76%) showed overestimation (Tables 3-4). The average overestimation was 14.97 ± 2.07%. The Ac optimized (V) calculation in this dataset shows an underestimation of the area in 38 out of 66 calculations (57.58%). The average underestimation was 7.29 ± 0.70%. Twenty-eight out of 66 calculations (42.42%) showed overestimation. The average overestimation was 13.24 ± 2.54%. The root mean square (RMS) errors of Ac standard and Ac elliptical are close to their averages but at the upper level (Tables 3-4). This means that standard and elliptical calculation consistently overestimates the area in these observations, but optimized calculations can lead to overestimation or underestimation. This may be because the optimization formula used here was developed for plantar and venous ulcers in human beings.

**Table 3:** Comparison of the methods for the overestimation and underestimation.

Orthogonal measurements (n=66)						
Method	Overestimation			Underestimation		
	Count	% Count	% Area	Count	% Count	% Area
Standard	100	100	48.09 ± 2.53	0	0	0
Elliptical	100	100	12.66 ± 1.98	0	0	0
Optimized (p)	50	75.76	8.65 ± 2.07	16	24.24	1.79 ± 0.64
Optimized (v)	28	42.42	6.58 ± 2.54	38	57.58	5.38 ± 0.70

**Table 4:** Error percentage of orthogonal measures in different calculations (n=66).

Error %/Method	Standard		Elliptical		Optimized (P)		Optimized (V)	
	Count	%	Count	%	Count	%	Count	%
0-9	0	0	24	36.36	43	65.15	43	65.15
10-19	0	0	20	30.30	9	13.64	17	25.76
20-29	4	6.06	8	12.12	8	12.12	2	3.03
30-39	20	30.30	8	12.12	2	3.03	2	3.03
40-49	16	24.24	1	1.52	2	3.03	2	3.03
50-59	9	13.64	1	1.52	1	1.52	0	0
60-69	9	13.64	3	4.55	1	1.52	0	0
70-79	2	3.03	1	1.52	0	0	0	0
80-89	1	1.52	0	0	0	0	0	0
90-99	1	1.52	0	0	0	0	0	0
100 and above	4	6.06	0	0	0	0	0	0

**Figure 1:**
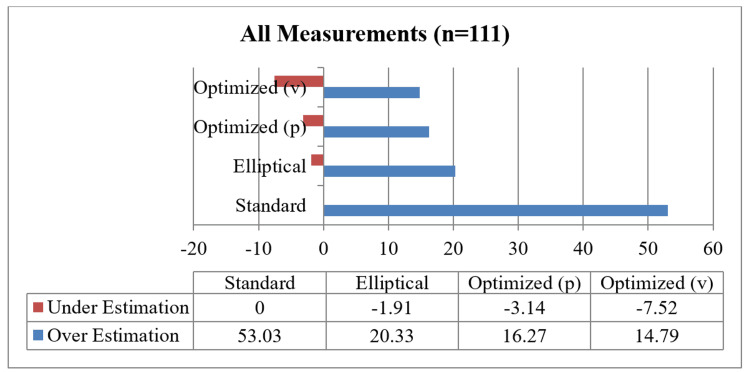
Over- or underestimation of area measurements (n=111).

The overestimation and underestimation of all the measurements (n=66) for orthogonal measurements and 45 for non-orthogonal measurements) were presented in Figure 1 for standard, elliptical, optimized (P), and optimized (V) measurement methods.

Cutaneous wounds with non-orthogonal measurements (n=45)

Forty-five (40.54% of the total) observations from different wounds of captive Asian elephants at different stages were collected for this study. The mean length, width, area, and perimeter are presented in Table 5. The Ac (standard) calculation in this dataset shows an overestimation of the area consistently. The average variation or mean RMS error was 55.45 ± 4.99%.

**Table 5:** Mean, median, SD, SE, and mean ± SE.

Non-orthogonal measurements (n=45)					
	Mean	Median	SD	SE	Mean ± SE
Length	8.387111111	5.98	6.956626214	1.037032607	8.39 ± 1.04 cm
Width	3.496888889	1.86	4.04255591	0.602628654	3.50 ± 0.60 cm
Area	33.05311111	6.62	65.10916727	9.705901599	33.05 ±9.71 cm^2^
Perimeter	20.43155556	12.29	19.45728339	2.900520555	20.43 ± 2.90 cm
Standard	50.59630444	9.394	109.6128236	16.34011498	50.60 ± 16.34 cm^2^
RMS error (Std)	55.45092638	45.61625	33.47986973	4.990884306	55.45 ± 4.99%
Elliptical	39.71809899	7.37429	86.04606651	12.82699026	39.72 ± 12.83 cm^2^
RMS error (Eli)	22.0289772	14.3087563	26.28169774	11.92828394	22.03 ± 11.93%
Optimized (P)	36.93530224	6.85762	80.01736122	3.91784418	36.94 ± 3.92 cm^2^
RMS error (Opt-p)	14.78302612	6.2998625	23.65673989	3.526538568	14.78 ± 3.53%
Optimized (V)	33.89952398	6.29398	73.4405918	10.94787704	33.90 ± 10.95 cm^2^
RMS Error (Opt-v)	12.23472508	8.48063793	19.17587496	2.858570662	± 2.89 %

The Ac (elliptical) calculation in this dataset shows an overestimation of the area consistently. The average variation or mean RMS error was 22.03 ± 11.93%. However, the Ac optimized (P) calculation in this dataset shows an underestimation of the area in 11 out of 45 calculations (24.44%). The average underestimation was 2.67 ± 0.63%. Thirty-four out of 45 calculations (75.56%) showed an overestimation. The average overestimation was 18.70 ± 4.47%. Ac optimized (V) calculation in this dataset shows an underestimation of the area in 23 out of 45 calculations (51.11%). The average underestimation was 7.91 ± 0.67%. Twenty-two out of 45 calculations (48.89%) showed an overestimation. The average overestimation was 16.76 ± 5.71%. The RMS errors of Ac standard and Ac elliptical are near to their averages, but at a higher level. This means that standard and elliptical calculation consistently overestimates the area in these observations, but optimized calculations can lead to an overestimation or underestimation. This may be because the optimization formula used here was developed for plantar and venous ulcers in humans.

For cutaneous wounds with non-orthogonal measurements, 45 (40.54% of the total) observations from different wounds of captive Asian elephants at different stages were collected for this study. The overestimation values for % wound area were noted to be 50.32 ± 4.99, 14.21 ± 11.93, 9.77 ± 4.47, and 8.87 ± 5.71 for standard, elliptical, optimized (p), and optimized (v) methods, respectively (Table 6). The error percentage for orthogonal measures in different calculations was calculated. The results indicated that orthogonal (V) measurements have the lowest error percentage across all ranges. No error percentage was found at 30-39% and 40-49% at orthogonal measurements. The elliptical measurements recorded the highest error percentages at 30-39% and 40-49%. The standard and optimized (P) methods are in between, with the optimized (P) value having a slight error percentage lower than the standard (Table 7).

**Table 6:** Comparison of methods for the overestimation and underestimation.

Orthogonal measurements (n=45)						
Method	Overestimation			Underestimation		
	Count	% Count	% Area	Count	% Count	% Area
Standard	100	100	50.32 ± 4.99	0	0	0
Elliptical	100	100	14.21 ± 11.93	0	0	0
Optimized (p)	34	75.56	9.77 ± 4.47	11	24.44	1.59 ± 0.63
Optimized (v)	22	48.89	8.87 ± 5.71	23	51.11	7.22 ± 0.67

**Table 7:** Error percentage of orthogonal measures in different calculations (n=45).

Error %/Method	Standard		Elliptical		Optimized (P)		Optimized (V)	
	Count	%	Count	%	Count	%	Count	%
0-9	0	0.00	14	31.11	23	51.11	27	60.00
10-19	0	0.00	14	31.11	11	24.44	13	28.89
20-29	2	4.44	6	13.33	6	13.33	4	8.89
30-39	12	26.67	6	13.33	4	0.00	0	0.00
40-49	9	20.00	4	8.89	0	0.00	0	0.00
50-59	8	17.78	0	0.00	0	0.00	0	0.00
60-69	5	11.11	0	0.00	0	0.00	0	0.00
70-79	4	8.89	0	0.00	0	0.00	0	0.00
80-89	4	8.89	0	0.00	0	0.00	0	0.00
90-99	0	0.00	0	0.00	0	0.00	0	0.00
100 and above	1	2.22	1	2.22	1	2.22	1	2.22

**Figure 2:**
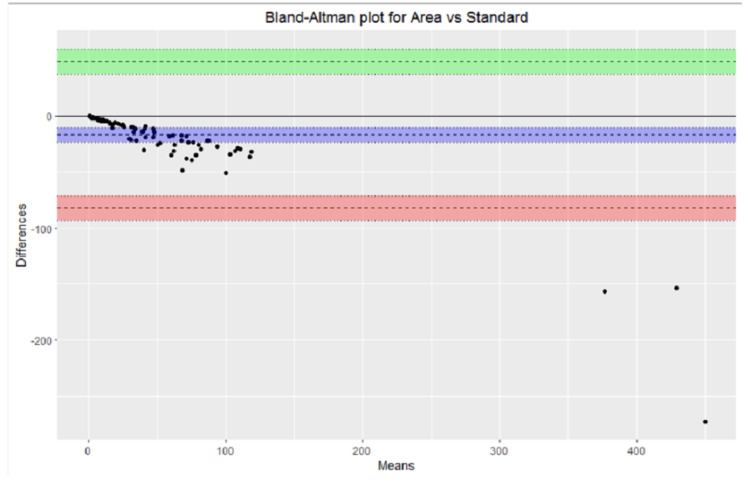
Bland-Altman plot area vs. standard.

**Figure 3:**
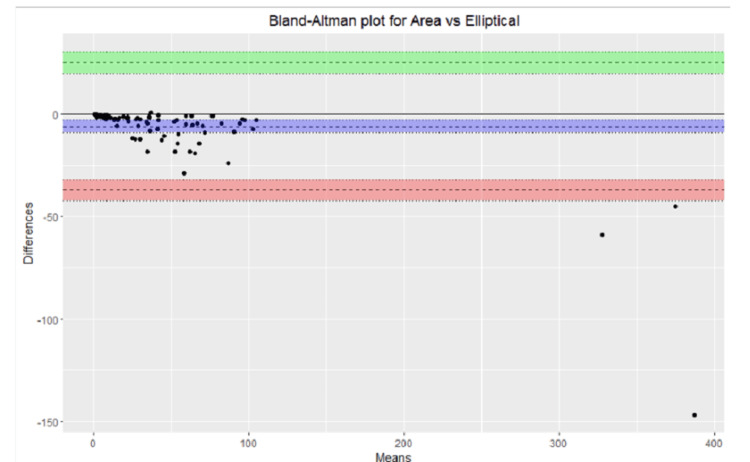
Bland-Altman plot area vs. ellipse.

**Figure 4:**
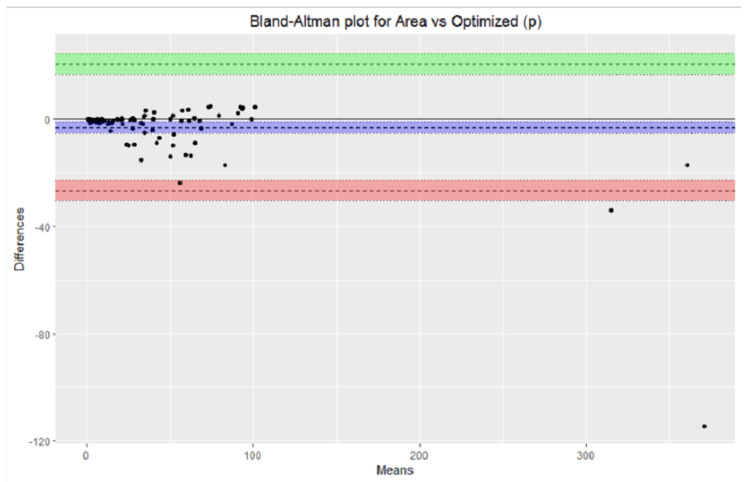
Bland-Altman plot area vs. optimized (p).

**Figure 5:**
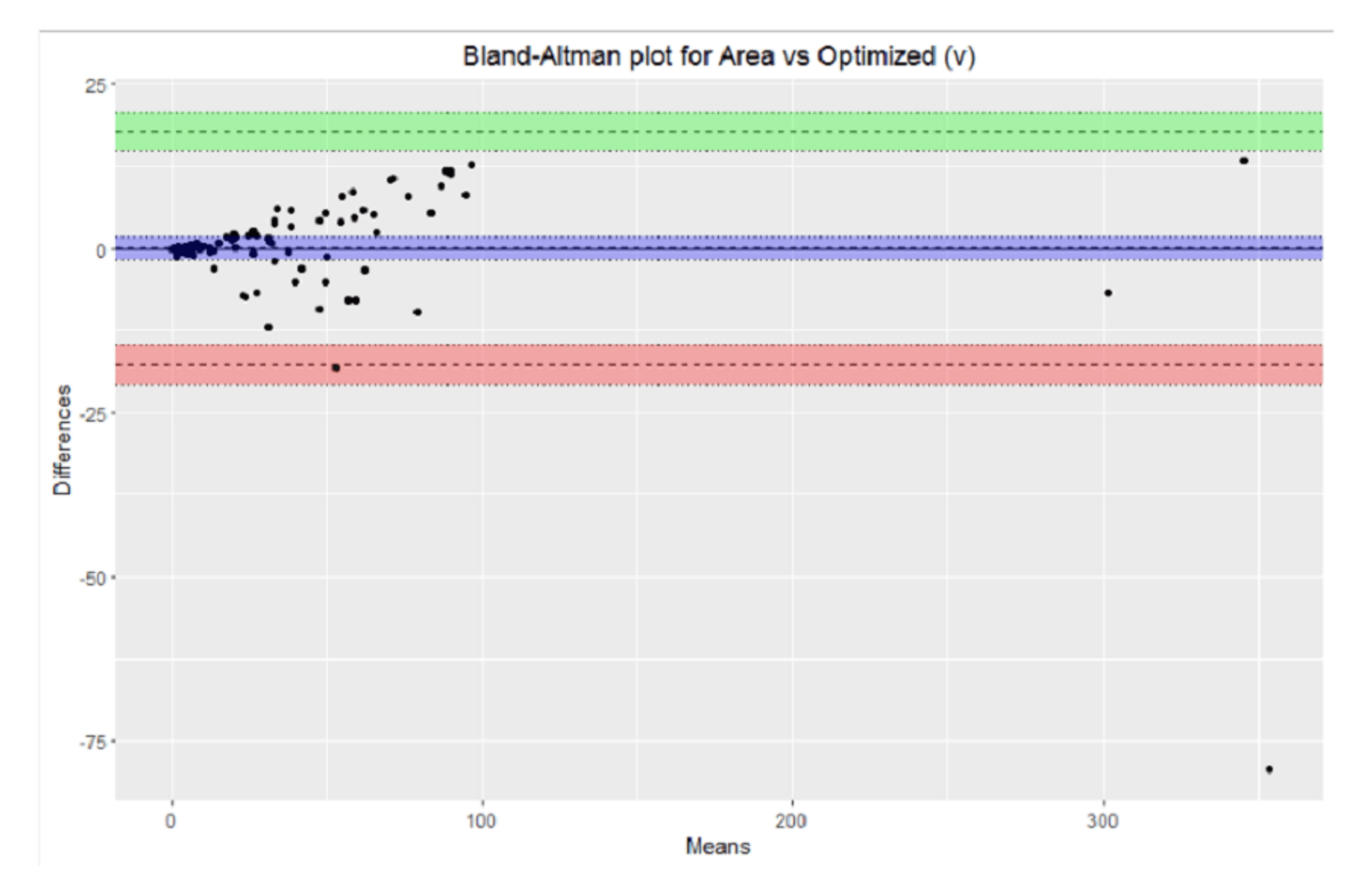
Bland-Altman plot area vs. optimized (v).

The Bland-Altman plots were used to represent the relationship between smartphone application measurement vs standard ruler, smartphone application measurement vs elliptical, smartphone application measurement vs. optimized (p), and smartphone application measurement vs. optimized (p) are shown in Figures 2-5.

Statistical analysis

One-way ANOVA was used to check the difference between various measurements. Independent two-sample t-tests were used for comparing measurement methods via orthogonal and non-orthogonal measurements. The one-way ANOVA study shows no statistically significant relations between the five types of measurement methods in the orthogonal and non-orthogonal measurement sections. The p-values were greater than 0.05, suggesting no significant correlation (Table 8). The t-test also demonstrated no significant relationship between the mean of the two groups (measurement methods via orthogonal and non-orthogonal measurements), as the P-value was greater than 0.05 (Table 9).

**Table 8:** One-way ANOVA test for the measurement methods.

Sl. No	Measurement methods	Area measurements (cm^2^), mean ± SE		
		All (n=111)	OM (n=66)	NOM (n=45)
1.	Imito Measure app (A)	34.61 ± 5.31	35.67 ± 4.65	33.05 ± 9.71
2.	Standard ruler (A_cs)_	51.60 ± 8.29	52.29 ± 6.59	50.60 ± 16.34
3.	Ellipse (A_ce_)	40.51 ± 6.51	41.05 ± 5.18	39.72 ± 12.83
4.	Optimized-p (A_cop_)	37.67 ± 1.97	38.17 ± 4.82	36.94 ± 3.92
5.	Optimized-v (A_cov_)	34.57 ± 5.56	35.03 ± 4.42	33.90 ± 10.94
6.	f-value	1.2013	1.1021	0.3181
7.	p-value	0.3092	0.3555	0.8657

**Table 9:** T-test for the measurement methods. Since the p-value is > 0.05, there is no significant difference between the five area measurement methods in all orthogonal and non-orthogonal measurements. OM-orthogonal measurements (n=66), NOM-non orthogonal measurement

Variable	Conditions	Mean	t-stat	p-value
Standard ruler	OM	52.29	-0.09	0.93
	NOM	50.60		
Ellipse	OM	41.05	-0.09	0.93
	NOM	39.72		
Optimized (p)	OM	38.17	-0.09	0.93
	NOM	26.94		
Optimized (v)	OM	35.03	-0.09	0.93
	NOM	33.90		

## Discussion

For venous and arterial cutaneous wounds, area measurement is suggested [14,15]. Just 5% of clinical trials regarding wound assessment in human wounds have addressed the validity or reliability of measuring methodologies [16]. The major methods employed in clinical studies are planimetry digital photography, computer software program analysis, direct measurement of wound diameters, specialized photographic software (using a smartphone application), and laser technology [17,18]. Although other approaches are available, computer software programs and planimetry have been the most widely used. The ideal approach would provide precision, dependability, and practicality simultaneously. When employed as a point-of-care tool, using a smartphone app promotes high feasibility [19]. Comparisons with a previously tested approach will assure consistency and interrater agreement [18].

Smartphones with high-definition digital cameras nowadays are readily available and reasonably priced. Such gadgets' exceptional portability and mobility are particularly inspiring for therapeutic applications. Imito and other smartphone-specific apps have arisen to make wound assessment and documentation easier and more straightforward [20]. The Imito app is a noncontact digital planimetry app with an edge over other approaches. When used to record, quantify, and evaluate metatarsal wound photos in a cat patient, the ImitoMeasure app found strong correlations across all studied metrics. Furthermore, the application enabled a noncontact, simple, and reliable smart wound-measuring solution [21].

For measuring the cutaneous wounds of elephants, 111 observations from 35 different wounds of captive Asian elephants at different stages were collected for study. The mean length was 8.45 ± 0.54 (Table 1), the mean width was 4.23 ± 0.34 (Table 1), the mean area was 34.61 ± 5.31 cm^2^, and the mean perimeter was noted to be 21.83 ± 1.51 cm. The overestimation value for the standard ruler method was 53.03%, while those of the other three methods were comparatively lower. When compared to digital planimetry, Rogers et al. discovered that the basic ruler approach underestimated the wound area by 41% on average [12]. Similarly, Shetty et al. [22] found that the simple ruler approach was overestimated by 29-43% compared to manual planimetry.

For cutaneous wounds with orthogonal measurements, 66 observations (59.46% of total observations) from different wounds of captive Asian elephants at different stages were collected for this study. The overestimation values for % wound area were noted to be 48.09 ± 2.53, 12.66 ± 1.98, 8.65 ± 2.07, and 6.58 ± 2.54 for standard ruler, elliptical, optimized (p), and optimized (v) methods, respectively. This may be because the optimization formula used here was developed for plantar and venous ulcers in humans. The plantar and palmar areas in humans are smaller in size compared to that of elephants.

For cutaneous wounds with non-orthogonal measurements, 45 (40.54% of the total) observations from different wounds of captive Asian elephants at different stages were collected for this part of the study. The overestimation values for % wound area were noted to be 50.32 ± 4.99, 14.21 ± 11.93, 9.77 ± 4.47, and 8.87 ± 5.71 for standard ruler, elliptical, optimized (p), and optimized (v) methods, respectively. This may be because the optimization formula used here was developed for the plantar and venous ulcers in human beings (Table 6).

Graphical representation provided a quick overview of the over and underestimation, as in Figure 1. Visual representation of the relationship between different measurement calculations against measurements done by the smartphone applications over the same scale done by Bland-Altman plots provided the ability to see a phenomenon but did not test it. In other words, it did not give us the same chance of error on a judgment regarding the variables that a test would do [23].

The observed results indicated no significant difference between the five methods of area measurement in all the studied cases (orthogonal and non-orthogonal measurements) since the p-value is > 0.05. However, published literature has demonstrated varied outcomes. For instance, Oien et al. examined four wound area assessment methods in 20 individuals with 50 chronic leg ulcers from various causes [24]. Digital planimetry, mechanical planimetry, grid tracing (manual planimetry), and a basic ruler approach were used. All approaches indicated a high level of concordance in wounds less than 10 cm^2^ (p < 0.01), although discrepancies emerged as wound size increased. It has previously been demonstrated that digital planimetry produces less variability than manual planimetry [25]. On the other hand, the Visitrak® system, which uses a tracing grid sheet for manual tracing and a digital pad for calculating the wound dimensions, revealed strong intra and interrater reliability, with a substantial positive correlation (p < 0.001) between the said system and the digital imaging approach when evaluated in 30 pressure ulcers [9].

The major strength of this study was that it was conducted in clinical cases. Hence, the wound areas under the study varied widely, from 0.09 cm^2^ to 352.14 cm^2^, which helped to test the feasibility of the proposed methods under study in a wide range of area measurements without any prejudice of the expected healing pattern of created wounds of known size. Another strong point about the study was that the same person did all the measurements to avoid individual variations in performing the measuring technique when more people were doing it. 

The study has the possible limitations as follows. The study was planned in such a way that the data collection should be non-contact and with the least animal discomfort. Hence, the possible level of animal discomfort in contact measuring techniques could not be assessed. Another thing is that this study compared only the mathematical calculations in order to check whether these formulas are dependable irrespective of the data source. The study did not assess the existing data collection methodologies in detail as it was beyond the scope of this study.

## Conclusions

If wound measurement techniques are to be beneficial for assessing the process of wound healing, they must be accurate, reliable, and feasible. The published studies revealed that wound measurement methods, including digital planimetry, are less validated by clinical trials. When utilized correctly, digital planimetry may be employed in wound area assessment and therapy evaluation, and the results indicate that smartphone digital planimetry has an advantage over existing procedures in terms of practical usability and electronic medical documentation. In animals, being a non-contact technique, this has an edge over other methods in terms of welfare concerns. Smartphones with high-definition digital cameras are now readily available, and their exceptional portability and mobility are especially promising for therapeutic applications. Smartphone-specific apps have arisen to make wound assessment and documentation more straightforward.

In this study, four traditional methods (mathematical calculation) applied in 111 wound area measurements are compared with a noncontact digital planimetry app, Imito Measure. The area of wounds ranged from 0.09 cm^2^ to 352.14 cm^2^. We found that all four mathematical methods overestimated (the highest being 53.03%) or underestimated (the highest being 7.52%) the wound area, consistent with previous studies. However, in all of the cases analyzed in the present study, the observed findings revealed no statistically significant difference between the five techniques of wound area measurement. From the practical clinical utility point, the smartphone application has the edge over the four mathematical methods used in this study, especially in captive Asian elephants, as it has the advantage of being non-contact and thus addresses some major welfare concerns. As suggested in the previous studies, assessment methods must be accurate, trustworthy, and practical for daily clinical work and clinical research investigations when comparing treatment regimens, and the ImitoMeasure app perfectly fits into this.
